# Improving Concordance Between Clinicians With Australian Guidelines for Bowel Cancer Prevention Using a Digital Application: Randomized Controlled Crossover Study

**DOI:** 10.2196/46625

**Published:** 2024-02-22

**Authors:** Tsai-Wing Ow, Olga Sukocheva, Peter Bampton, Guruparan Iyngkaran, Christopher K Rayner, Edmund Tse

**Affiliations:** 1 Department of Gastroenterology and Hepatology Royal Adelaide Hospital Adelaide Australia; 2 Faculty of Health and Medical Sciences University of Adelaide Adelaide Australia; 3 Department of Gastroenterology and Hepatology Royal Melbourne Hospital Melbourne Australia

**Keywords:** colorectal cancer, guidelines, colorectal cancer screening, digital application, questionnaire, application, cancer prevention, prevention, cancer, bowel cancer, surveillance, clinical vignette quiz, usability, Australia

## Abstract

**Background:**

Australia’s bowel cancer prevention guidelines, following a recent revision, are among the most complex in the world. Detailed decision tables outline screening or surveillance recommendations for 230 case scenarios alongside cessation recommendations for older patients. While these guidelines can help better allocate limited colonoscopy resources, their increasing complexity may limit their adoption and potential benefits. Therefore, tools to support clinicians in navigating these guidelines could be essential for national bowel cancer prevention efforts. Digital applications (DAs) represent a potentially inexpensive and scalable solution but are yet to be tested for this purpose.

**Objective:**

This study aims to assess whether a DA could increase clinician adherence to Australia’s new colorectal cancer screening and surveillance guidelines and determine whether improved usability correlates with greater conformance to guidelines.

**Methods:**

As part of a randomized controlled crossover study, we created a clinical vignette quiz to evaluate the efficacy of a DA in comparison with the standard resource (SR) for making screening and surveillance decisions. Briefings were provided to study participants, which were tailored to their level of familiarity with the guidelines. We measured the adherence of clinicians according to their number of guideline-concordant responses to the scenarios in the quiz using either the DA or the SR. The maximum score was 18, with higher scores indicating improved adherence. We also tested the DA’s usability using the System Usability Scale.

**Results:**

Of 117 participants, 80 were included in the final analysis. Using the SR, the adherence of participants was rated a median (IQR) score of 10 (7.75-13) out of 18. The participants’ adherence improved by 40% (relative risk 1.4, *P*<.001) when using the DA, reaching a median (IQR) score of 14 (12-17) out of 18. The DA was rated highly for usability with a median (IQR) score of 90 (72.5-95) and ranked in the 96th percentile of systems. There was a moderate correlation between the usability of the DA and better adherence (*r*_s_=0.4; *P*<.001). No differences between the adherence of specialists and nonspecialists were found, either with the SR (10 vs 9; *P*=.47) or with the DA (13 vs 15; *P*=.24). There was no significant association between participants who were less adherent with the DA (n=17) and their age (*P*=.06), experience with decision support tools (*P*=.51), or academic involvement with a university (*P*=.39).

**Conclusions:**

DAs can significantly improve the adoption of complex Australian bowel cancer prevention guidelines. As screening and surveillance guidelines become increasingly complex and personalized, these tools will be crucial to help clinicians accurately determine the most appropriate recommendations for their patients. Additional research to understand why some practitioners perform worse with DAs is required. Further improvements in application usability may optimize guideline concordance further.

## Introduction

Australia’s National Health and Medical Research Council (NHMRC) screening and surveillance guidelines for colorectal cancer have become substantially more complex with their latest revision [1,2]. This is due to a shift toward personalized recommendations through detailed risk stratification based on an individual’s history of polyps or a family history of cancer. As a result, the guidelines now describe up to 230 different screening or surveillance scenarios, requiring clinicians to navigate through multiple tables to determine an appropriate recommendation. While implementing these changes can considerably improve resource use, this complexity may be a barrier to adherence, limiting the benefits of the guidelines [3,4]. Consequently, there is a need for tools to support clinicians using these guidelines. However, few of these tools have been adequately evaluated.

Several approaches have previously been considered to assist clinicians in determining appropriate bowel cancer prevention guideline recommendations. In the United States, where the complexity of polyp surveillance guidelines is the most similar to those of Australia, researchers have primarily focused on developing methods to assist clinicians in determining the appropriate advice, with a particular emphasis on automating the extraction of clinical information from electronic records to determine guideline-concordant recommendations [5-7]. In clinical practice, this resulted in a small but significant improvement in the rate of guideline-concordant recommendations (84.6% vs 77.4%) [7]. In Australia, print-based educational interventions for screening and surveillance, targeted at patients and clinicians, respectively, have had a minimal impact on improving guideline adherence [8,9]. By contrast, a nurse-led decision-making model has been the most successful intervention, increasing the rate of guideline-concordant recommendations from 83% to 97% [10]. Although successful, these options are associated with substantial costs for setup and maintenance and are not easily scalable beyond individual health services. Furthermore, how they perform when applied to the recently revised Australian guidelines is unclear.

Smartphone- or web-based digital applications (DAs) can be developed cheaply and are readily scalable. However, there are limited studies evaluating their effectiveness in supporting clinician adherence to complex bowel cancer prevention guidelines. Khan et al [11] showed that a DA was able to improve medical students’ knowledge of US colorectal cancer screening guidelines. However, their study was not randomized and did not control for the improvement in scores merely due to repeated exposure to the same clinical questions. In another study, a DA was evaluated by 6 endoscopists assessing a total of 58 colonoscopies [12]. As this was a small pilot study primarily focused on assessing the attitudes of potential users to guide the development of a new DA, it is difficult to draw meaningful conclusions about the potential benefit of the tool in improving guideline concordance.

In Australia, some of the DAs developed in response to the complexity of the latest surveillance guidelines include polyp.guide, polyp.app, and CRCwebapp [13-15]. These 3 tools provide greater ease of use by not requiring users to work through the risk tables manually. To the best of our knowledge, only CRCwebapp has been validated against all 230 possible case scenarios due to its use as a research tool in a previously published study [3]. However, none of these have been evaluated for their ability to improve the rate of guideline concordance among clinicians.

We hypothesized that a DA could improve clinician adherence to Australian screening and surveillance guidelines. To test this, we conducted a randomized controlled crossover study to compare the proportion of guideline-concordant decisions made by clinicians using either the CRCwebapp DA or the standard resource (SR).

## Methods

### Study Design and Setting

We enrolled practicing Australian clinicians to our online randomized controlled crossover clinical vignette questionnaire between July 1, 2020, and August 1, 2021. Participants were asked to provide guideline-concordant recommendations for 2 sets of clinical vignettes using either the SR or the DA. All participants were provided with an orientation that was tailored according to their experience with the guidelines. The clinical vignettes and order in which the tools were used were randomized. A study portal was used to present the vignettes, and this provided participants with access to both the SR and DA. After completing questions related to the clinical vignettes with both the SR and DA, the System Usability Scale (SUS) questionnaire was administered.

### Inclusion Criteria

We included medical, surgical, or specialist nurse practitioners who were actively practicing in Australia during the study period.

### Exclusion Criteria

Participants who were not actively involved in making screening or surveillance decisions for colorectal cancer in their clinical work were excluded.

### Participant Orientation

We classified participants into 2 groups according to their familiarity with the guidelines. The nonspecialist group comprised primary care practitioners who had limited experience with the terminology and structure of the published guidelines. The specialist group comprised gastroenterologists, colorectal surgeons, and specialist nurse practitioners who were routinely using the current screening and surveillance guidelines in clinical practice. The orientation program was tailored according to the experience of each group, in order to reduce the impact of experience on participant scores and to reduce barriers to participation.

For nonspecialists, the necessary terminology pertaining to screening and surveillance was defined during a web seminar. This included degree of relationship in family history for screening protocols and the individual risk characteristics and classification of lesions for surveillance protocols. The seminar also included a breakdown of every decision table in the SR and the most efficient methods to navigate to each of these. Participants were also introduced to the 4 main pages of the DA and shown how to input data and where the results were presented. In contrast, the specialist orientation did not define the terminology, and the introductions to the SR and the DA were presented as optional videos available before the questionnaire.

### Primary Outcome

The primary outcome was the proportion of correct screening and surveillance recommendations issued by participants in response to the clinical vignettes. Each vignette could receive a maximum score of 6, resulting in each participant being graded with a score out of 18 for each of 2 sets of 3 clinical vignettes.

### Secondary Outcome

The secondary outcome was the usability of the DA. This was assessed using each participant’s response to the SUS. A score was determined for each participant and normalized in accordance with previously published methods [16].

### Clinical Vignette Design

Three pairs of clinical vignettes were developed for the study (alpha and beta, gamma and theta, and delta and omega). Each vignette described the family history, medical comorbidities, and the number and characteristics of conventional adenomas or sessile serrated lesions identified over the preceding 2 colonoscopies. We avoided scenarios commonly highlighted in previous guidelines to reduce the likelihood that participants could answer according to their recollection of these [17]. Each pair of clinical vignettes focused participants on navigating identical sets of tables to balance for difficulty.

For each clinical vignette, participants were asked to determine the age and appropriate screening modality (stool testing or colonoscopy) based on the family history presented, the first and subsequent recommended surveillance intervals, and whether surveillance should be continued when considering the comorbidities of the patient if the age of the patient was >75 years at the time of the intended procedure. Each vignette received a score out of 6. Thus, each participant could receive a maximum score of 18 for each section.

### Usability

We adapted the standard SUS questionnaire by changing the term “system” to “application” in order to focus participants on assessing the usability of the DA (Textbox 1). This comprised 10 standardized statements for which users were asked to indicate their level of agreement. Numerical scores provided by participants on a slider scale were translated into Likert scores: 0-20=strongly disagree (1); 21-40=disagree (2); 41-60=neither agree nor disagree (3); 61-80=agree (4); and 81-100=strongly agree (5). A total SUS score was calculated for each participant [18]. The scores were normalized to provide a percentile ranking of the usability of the DA, as described by Sauro and Lewis [16].

**System Usability Scale questionnaire adapted for the use of the digital application.**

1. I think that I would like to use this application frequently.
2. I found the application unnecessarily complex.
3. I thought the application was easy to use.
4. I think that I would need the support of a technical person to be able to use this application.
5. I found that the various functions in this application were well integrated.
6. I thought there was too much inconsistency in this application.
7. I would imagine that people would learn to use this application very quickly.
8. I found the application very cumbersome to use.
9. I felt very confident using the application.
10. I needed to learn a lot of things before I could get going with this application.

### DA Design

Each NHMRC screening and surveillance recommendation was coded into an Excel (Microsoft Corp) spreadsheet. We eliminated redundant user data entry by determining the minimum number of inputs necessary to calculate each recommendation. For screening decisions, this included 4 fields relating to the number and age of relatives with colorectal cancer and their relation (first or second degree) to the patient. For surveillance intervals, this included the number, type, and characteristics of the lesions found during the initial procedure. Subsequent surveillance intervals required 2 additional inputs: the initial surveillance interval and the type of lesion previously identified. An additional section, incorporating a list of potential patient comorbidities, was used to determine stopping rules.

A graphical user interface was applied using an open-source platform (Open as App), which would allow for the distribution of the DA as either a web page or smartphone app. Each type of calculation (screening, first surveillance, second surveillance, or stopping rules) was identified by a tab on the bottom of the screen. Sliders were used to input data on the number of lesions, and drop-down menus were used to provide details regarding the accompanying risk characteristics. The recommendations for screening, surveillance interval, or cessation of surveillance were provided at the bottom of each respective page. The answers provided by the digital calculator were validated by individually calculating all possible scenarios covered by the updated guidelines before recruitment.

### SR for Screening and Surveillance

The SR was the official web publication of the latest guidelines for screening and surveillance for bowel cancer prevention in Australia by the NHMRC [1,2]. In addition to a written summary, it provides details regarding the development of and evidence for each recommendation. Also included are a series of colored risk stratification tables to guide users through screening, initial and follow-up surveillance, and stopping rules. For screening, 90 possible scenarios are defined according to the number of relatives with colorectal cancer as well as how closely they are related to the patient.

For initial surveillance colonoscopy, 37 separate scenarios are described across 3 tables according to the various combinations of “conventional adenomas” or “clinically significant serrated polyps” identified. A total of 140 scenarios are similarly characterized across an additional 9 tables to account for the possible combinations of “conventional adenomas” and “clinically significant serrated polyps” between 2 consecutive procedures. Determining the correct surveillance interval can thus require users to successfully navigate 2 consecutive tables.

Lastly, the rules for cessation of surveillance colonoscopy are detailed in a text table that uses a modified Charlson score. Scores are allocated according to age and the presence of comorbidities. Depending on the combination of age and severity of comorbid conditions, the benefit of continuing surveillance for patients may be deemed too low to justify the potential risks of colonoscopy.

### Recruitment

Advertising flyers were created and distributed to the 3 local Primary Health Networks, social media (Facebook: Adelaide GP Referral Network, Medical Mums, and Mums To Be), general practitioner education providers (GPEx and GP Synergy), and directly to practice managers located within metropolitan Adelaide. Additional flyers for specialists were distributed to members of the Departments of Gastroenterology and Hepatology and Colorectal Surgery Departments at 4 major teaching hospitals in Adelaide, as well as to private specialist practices. Snowball sampling was used to aid in the recruitment of additional participants. Continuing professional development points and a certificate of completion were awarded as an incentive to improve recruitment.

### Data Collection

The questionnaire was programmed using REDCap (Research Electronic Data Capture; Vanderbilt University) tools hosted at the University of Technology, Sydney, and accessed through the Australian Access Federation [19,20]. We collected data including each participant’s age, professional background (general practice, medical specialist or trainee, and surgical specialist or trainee), active affiliation with a university, and experience with tools supporting screening and surveillance guidelines. We scored the answers for each clinical vignette in the order in which they were completed and collected each participant’s responses to the SUS questionnaire regarding their experience with the DA on a digital spreadsheet for analysis according to a previously described methodology [15].

### Randomization

Two randomly permuted schedules (primary care and specialist groups) were created for a crossover study with 2 interventions (DA vs SR) with equal allocation over 8 strata (combinations 1-8; Table 1). A total of 14 allocations were generated per stratum with a total of 112 allocations. Participants were randomized to use either the DA or SR as the first aid in a 1:1 ratio. The 2 allocation schedules were programmed into the REDCap software using branching logic tools. The randomization schema was generated using Microsoft Excel (version 16.66.1; Microsoft Corp).

**Table 1:** Clinical vignette combinations used for randomization.

Combination	Section 1	Section 2
1	Alpha, gamma, and delta	Beta, theta, and omega
2	Alpha, gamma, and omega	Beta, theta, and delta
3	Alpha, theta, and delta	Beta, gamma, and omega
4	Alpha, theta, and omega	Beta, gamma, and delta
5	Beta, gamma, and delta	Alpha, theta, and omega
6	Beta, gamma, and omega	Alpha, theta, and delta
7	Beta, theta, and delta	Alpha, gamma, and omega
8	Beta, theta, and omega	Alpha, gamma, and delta

### Statistics

Previously reported rates of adherence to Australian surveillance guidelines have ranged from 50.8% to 83% [4,10]. The impact of a nurse-led intervention improved the rate of guideline concordance by a factor of 1.17 relative to the non–nurse-led group [10]. On the basis of these results, we predicted a mean accuracy score of 60% with the SR and anticipated a 1.17 improvement in the rate of guideline concordance to 70% with the intervention (DA). Using an expected SD of 20%, an α of .05, and a statistical power of 0.8, the minimum necessary sample size required was calculated at 64 participants.

Descriptive statistics were used to characterize the data. A Kolmogorov-Smirnov test was applied to assess for normality of the data before the statistical analysis. A related-samples Wilcoxon signed rank test was used to compare the performances of participants with either the SR or the DA. An independent-samples Mann-Whitney *U* test was used to compare outcomes between specialists and nonspecialists. Spearman ρ was used to assess the relationship between usability and scores from the DA. *χ*^2^ tests of independence were used to compare the allocation of participants between tools and clinical vignettes. The SPSS statistical software (version 22; IBM Corp) was used for all analyses.

### Ethical Considerations

The study protocol was reviewed and approved by the Central Adelaide Local Health Network Human Research Ethics Committee (CALHN Research Office reference 13438). The background, procedures, and aims of the study were provided to prospective participants via a digital participant information sheet before the commencement of the survey. Participants were informed that their consent to participate would be implied via completion and submission of the online questionnaire. All data collected were deidentified. No participants received financial compensation.

## Results

### Participant Characteristics

**Figure 1:**
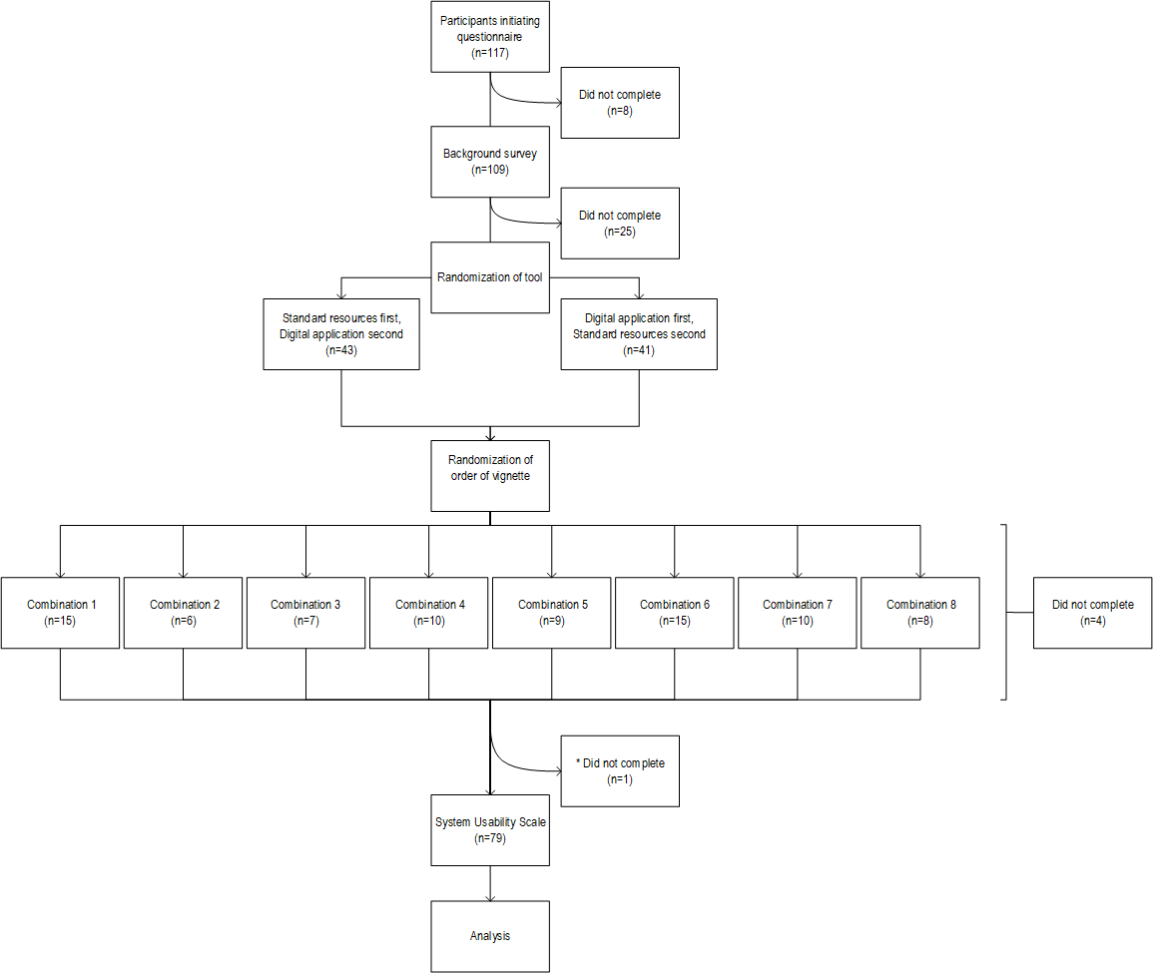
Study flowchart. *Included in the primary analysis but excluded from usability evaluation.

In total, 117 participants initiated the questionnaire. The records of 37 participants were excluded from the primary analysis due to survey noncompletion. Of these, no components of the questionnaire were attempted in 8 cases, 25 participants completed the background survey but did not attempt the clinical vignette section, and 4 participants aborted the clinical vignette section before completion (Figure 1). These included 7 primary care doctors, 20 gastroenterologists, 1 surgeon, 1 nurse endoscopist, and 8 participants of unknown vocation. One additional participant aborted the study after completing the vignettes and was included in the primary analysis but not in the evaluation of the usability scores.

**Figure 2:**
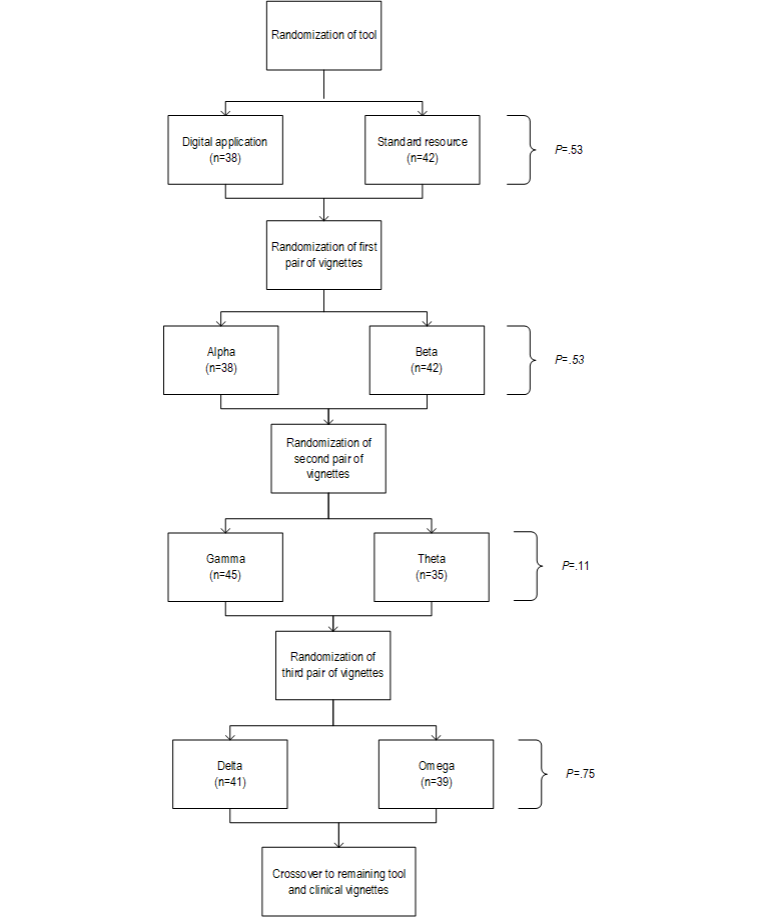
Allocation of tools and vignettes for the first set of clinical vignettes after randomization. Pearson χ2 tests of independence were used to assess the distribution order of tools (standard resource or digital application) and vignettes (alpha or beta, gamma or theta, and delta or omega) after excluding participants who did not complete the study. The analysis confirmed that the differences in the final allocation of participants at each stage after exclusions were not significant.

The remaining 80 participants, consisting of 43 primary care doctors and 37 specialist doctors (35 gastroenterologists and 2 surgeons), were included in the primary analysis. They had a median age of 38 (IQR 27-71) years. Fewer than half (35/80, 44%) held an affiliation with a university (27/37, 73% of specialists and 8/43, 19% of primary care doctors), and almost two-thirds (51/80, 64%) had previously used tools for screening and surveillance decisions in colorectal cancer (32/37, 87% of specialists and 19/43, 44% of primary care doctors; Table 2). The study flowchart shows how participants were randomized to 1 of 8 sequences of vignettes (Figure 1). Of the 80 included participants, 38 (48%) were assigned to use the DA as the first aid (Figure 2). Alpha, gamma, and delta were the first vignettes in their respective pairs in 48% (38/80), 56% (45/80), and 51% (41/80) of cases (Figure 2).

**Table 2:** Tools to aid decisions in colorectal cancer screening and surveillance (N=80).

Tool	Specialist (n=37), n (%)	Primary care (n=43), n (%)
Wiki.cancer Guideline (NHMRC^a^)	20 (54)	11 (26)
Polyp.guide	8 (22)	3 (7)
Digital calculator	6 (16)	1 (2)
Media in endoscopy suite	19 (51)	—^b^
Polyp nurse support	3 (8)	—
Funding codes (Medicare)	2 (5)	—
The Royal Australian College of General Practitioners’ Redbook	—	6 (14)
Other	1 (3)	2 (5%)

^a^NHMRC: National Health and Medical Research Council.

^b^Not available.

**Figure 3:**
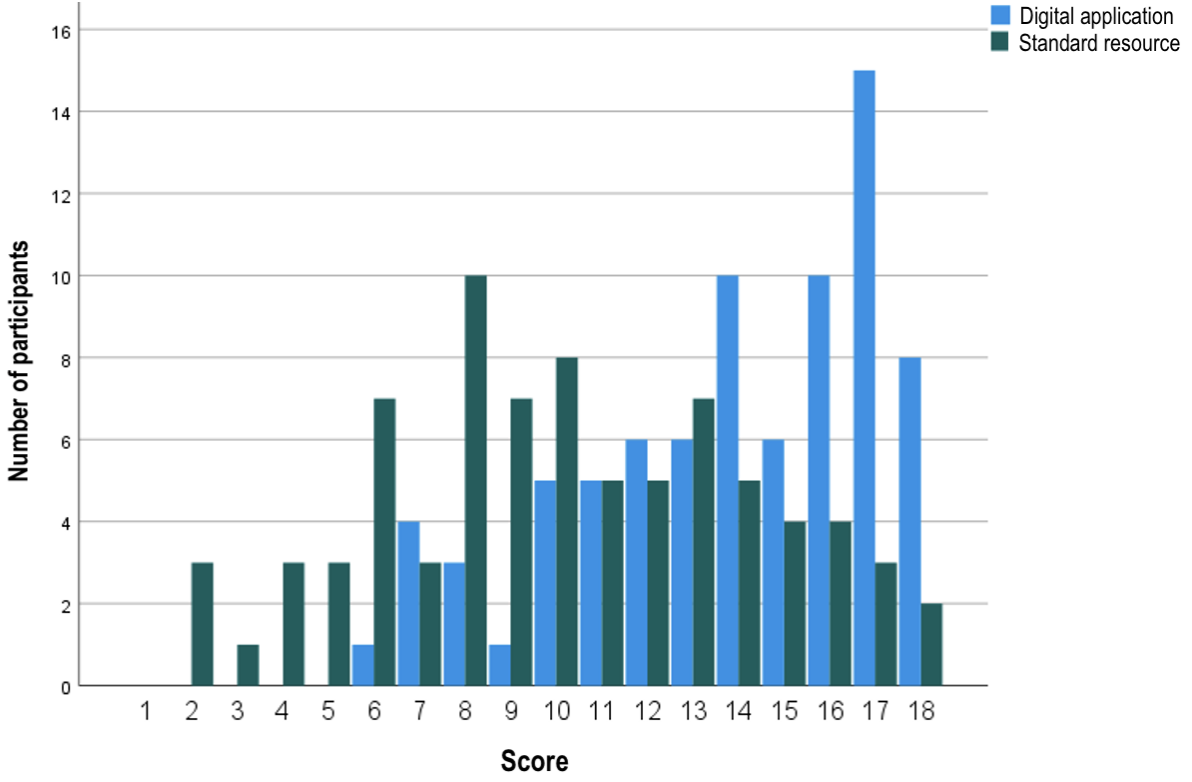
Comparison of spread of clinical vignette scores with either the standard resource (SR) or the digital application (DA). The participant scores when using the SR showed a normal distribution. A rightward shift in the distribution of the scores was observed with the use of the DA.

**Figure 4:**
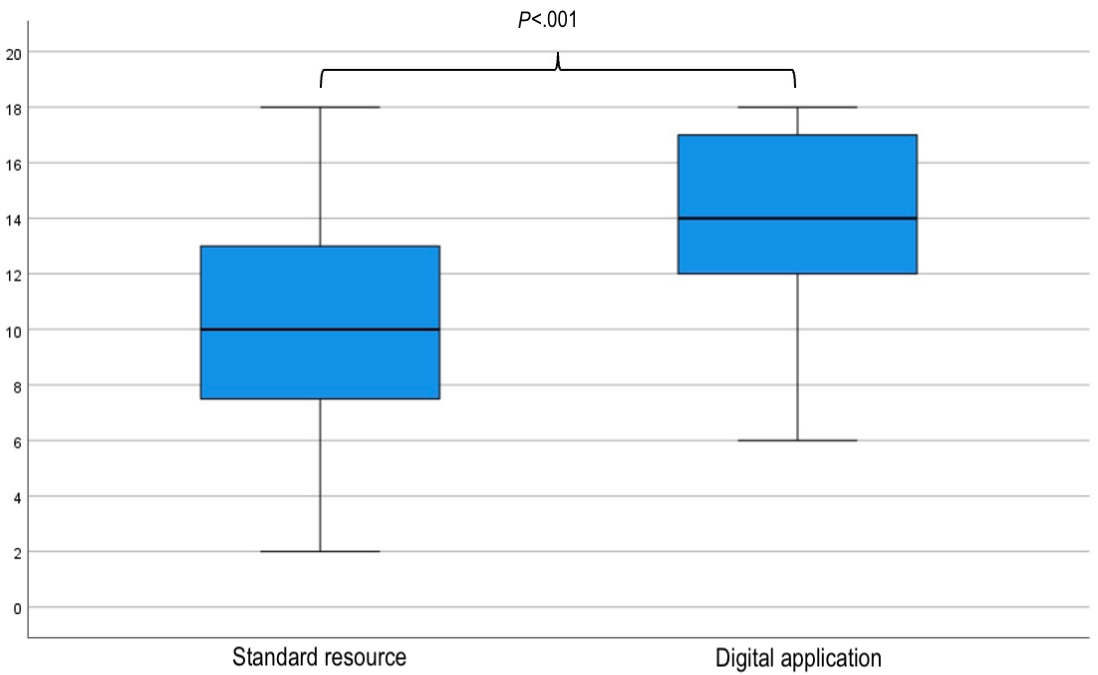
Box and whisker plot of clinical vignette scores with either the standard resource or the digital application.

A Kolmogorov-Smirnov test of normality indicated that the scores of participants using the SR were normally distributed: D(80)=0.075; *P*=.20, while those of the DA were not: D(80)=0.152; *P*<.001. With the SR, the median (IQR) number of guideline concordant answers was 10 (7.75-13) out of 18. The use of the DA improved the number of correct recommendations to a median (IQR) of 14 (12-17) out of 18 (relative risk 1.4, *P*<.001; Figures 3 and 4). Lower performance with the DA compared with SR (n=17) was not associated with previous experience with screening and surveillance decision tools (*P*=.51), affiliation with a university (*P*=.39), or age (*P*=.06).

The median (IQR) SUS score for the DA was 90 (72.5-95), which equated to a top 4 percentile ranking among tested applications (Table 3). A moderate correlation between usability grade and DA results was observed using Spearman ρ correlation coefficient (*r*_s_=0.4; *P*<.001; n=79).

**Table 3:** System Usability Scale (SUS) grades and percentiles for participants using the digital application (n=79).

Grade	SUS	Participants, n (%)	Percentile
A	>78.8	51 (65)	85-100
B	72.6-78.8	7 (9)	65-84
C	62.7-72.5	11 (14)	35-64
D	51.7-62.6	6 (8)	15-34
F	0-51.7	4 (5)	0-15

### Sensitivity Analysis

**Figure 5:**
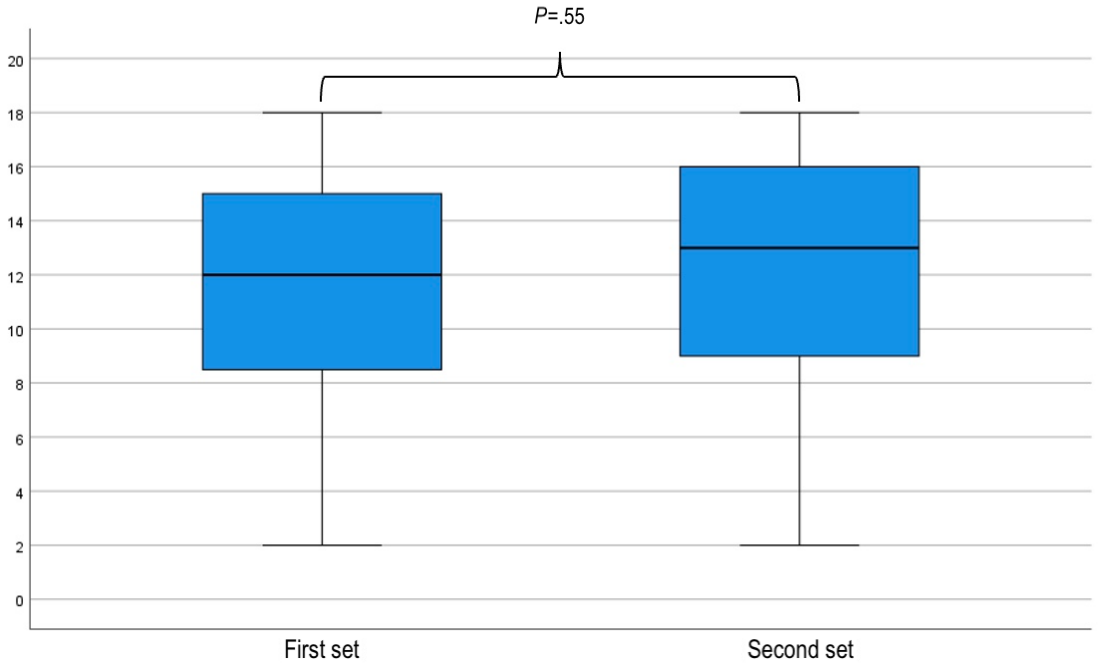
Box and whisker plot of clinical vignette scores according to the order they were answered (first or second). A related-samples Wilcoxon signed rank test was used to compare the results achieved in the first and second set of questions indicating no significant difference (*P*=.55). Thus, increasing familiarity with the format of the questionnaire did not improve the scores achieved by participants.

**Figure 6:**
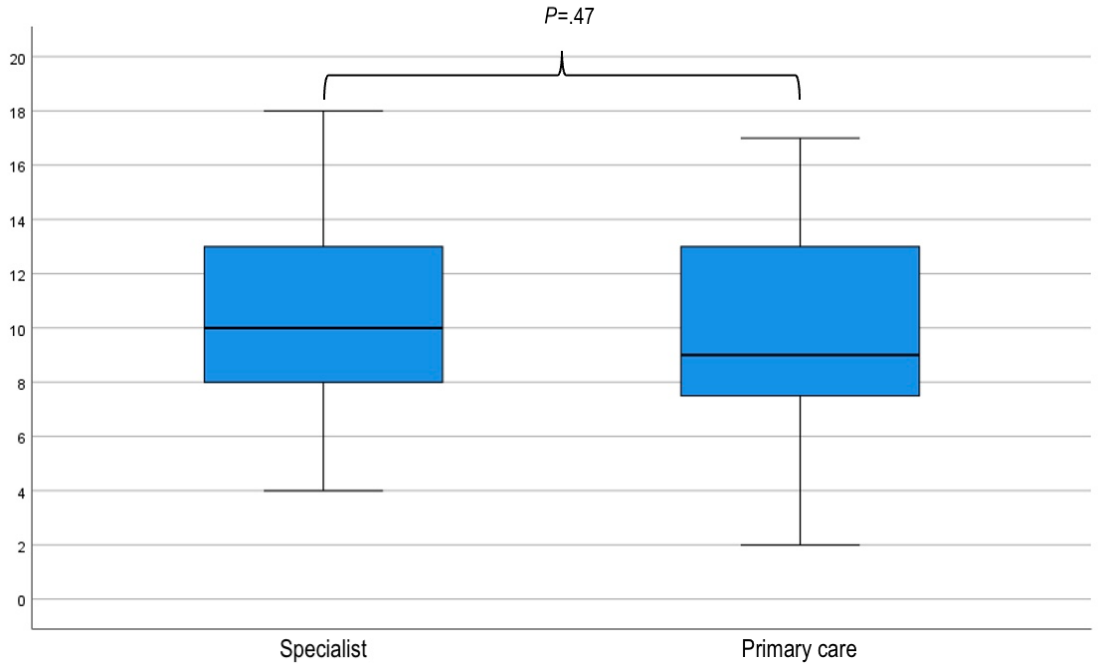
Box and whisker plot of clinical vignette scores according to the vocational training of the participants (specialist or primary care) using the standard resource (SR). An independent-samples Mann-Whitney U test was used to compare the results of specialists with primary care doctors using the SR. There was no significant difference (*P*=.47) in the performance of participants based on their previous training in either specialist or primary care.

**Figure 7:**
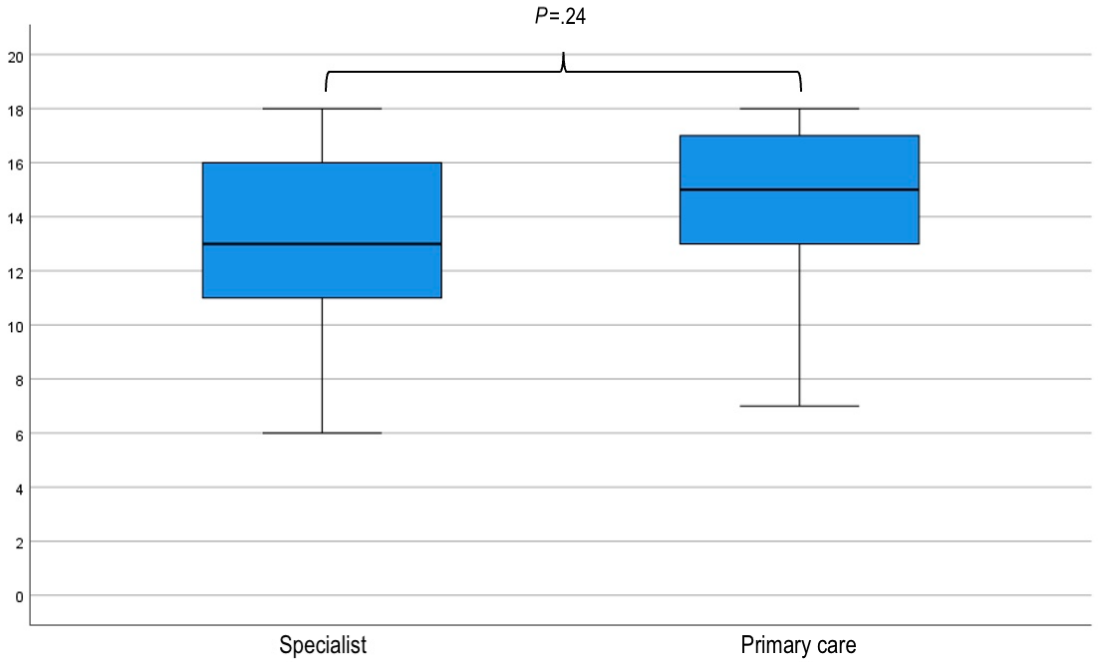
Box and whisker plot of clinical vignette scores according to the vocational training of the participants (specialist or primary care) using the digital application (DA). An independent-samples Mann-Whitney U test was used to compare the results of specialists with primary care doctors using the DA. There was no significant difference (*P*=.24) in the performance of participants based on their previous training in either specialist or primary care.

After excluding those who did not complete the study, differences in the randomization of participants regarding order of use of the tools (SR vs DA) and clinical vignettes (alpha vs beta, gamma vs theta, and delta vs gamma) were not significant (Figure 2). Additionally, there was no difference (*P*=.55) between the median number of guideline concordant recommendations according to whether the clinical vignettes were posed to participants: first (12, IQR 8.75-15) or second (13, IQR 9-16; Figure 5). Similarly, no difference was observed between the performance of specialists and primary care doctors, either with the SR (10 vs 9; *P*=.47) or with the DA (13 vs 15, *P*=.24; Figures 6 and 7).

## Discussion

### Principal Findings

The findings of this study showed that the adherence of clinicians with Australia’s current screening and surveillance guidelines in their current form is limited. This was significantly improved when clinicians used a DA to assist their navigation of these complex guidelines. These findings were independent of the clinicians’ level of specialization, age, university affiliation, or experience with the use of other decision support tools. However, greater adherence was associated with better DA usability ratings, highlighting the importance of this attribute as a potential target to further bolster clinician guideline adherence.

Australia’s screening and surveillance guidelines are among the most complex worldwide. With the increasing trend toward personalized health care and our growing knowledge of colorectal cancer risk factors, guidelines are likely to continue increasing in complexity. For clinicians, navigating these guidelines in busy practices can be challenging. Even under the controlled conditions of our testing environment, participants could only provide appropriate recommendations in slightly over half of the questions when evaluating the scenarios only with the SR. These findings are consistent with another recent report that assessed the concordance of surveillance recommendations with current guidelines [4]. Because the adherence to previous relatively more straightforward guidelines was already known to be suboptimal, it could be anticipated that rates of adherence may be even lower as their complexity increases. This could undermine their potential benefits in the care of patients and the allocation of limited colonoscopy resources in Australia.

DAs can play an important role in supporting the implementation of Australia’s complex bowel cancer prevention guidelines. Not only do they improve the ability of clinicians to provide guideline-concordant recommendations, as demonstrated by our study, but they can be developed at a relatively low cost and are scalable to a national level. Furthermore, they can be updated with future revisions of the guidelines, ensuring that clinicians can continue to make decisions that are in keeping with the latest evidence.

Despite their clear advantages, the role of DAs in supporting complex guideline adoption has received little attention in the literature. To date, only 2 studies have evaluated DAs in assisting medical personnel with the application of bowel cancer screening and surveillance guidelines. However, these were assessed in relation to US guidelines and are limited by their small size and lack of a randomized controlled methodology. To our knowledge, our study is the first to evaluate a DA using a rigorous randomized controlled crossover design.

Participants provided discordant recommendations in 22% of clinical decisions despite assistance from the DA. However, as the DA used in this study had been validated across all the possible scenarios provided by the guidelines, we considered other factors that may have contributed to this. Our results showed that poor performance with the DA relative to the SR was not associated with participant age, academic experience, or prior experience with similar tools. One area that may have contributed was DA usability. Although the DA scored very well in the SUS, ranking at or above the 96th percentile of tested systems, there was still a relatively large spread of scores (median 90, IQR 72.5-95) and a moderate correlation between SUS scores and participant performance. This suggests that improvements directed at improving usability for those who scored the DA less well could bolster the adherence rate of clinicians with guidelines; however, the magnitude of overall improvement may be small. Therefore, additional research to gather the opinions of participants who found the interface difficult to use and quantify the degree of progress achieved by addressing these is required.

Human error is another potential factor contributing to the rate of discordant answers. Despite simplifying the process of determining guideline-concordant recommendations, the DA still requires individuals to extract relevant and appropriate data from sometimes complex patient histories. Although human error remains an inevitable component of any interface requiring human input, natural language processing software, which has been used in prior US-based studies, could provide a valuable adjunct to a mobile app [5,6]. This would retain the scalability and portability of the DA but would require additional research, development, and testing before it could be implemented. Such a tool could provide a better balance of the advantages of the tools tested thus far.

### Strengths

Our study design accounted for the possibility that participants could improve their performance in the clinical vignettes simply due to increasing experience with the questionnaire, by randomizing the order of use of the 2 aids (SR or DA). Furthermore, although the clinical vignettes were designed in pairs that were balanced for difficulty, the order in which each pair was presented to the participant was randomized to limit the risk of bias. The vignettes also focused on clinical scenarios with updated and distinct recommendations within the guidelines, requiring participants to determine the correct answer solely through navigation of the SR or DA.

Another strength of our study was the ability to cater for participants with varying levels of familiarity with guidelines. Our participants included specialists, who are accustomed to using colorectal cancer screening and surveillance guidelines in their everyday practice, and nonspecialists, whose breadth of clinical practice typically limits their experience with specialty guidelines. As these differences may have impacted participant vignette questionnaire scores, particularly those encountered without the DA, we tailored the introductory briefings to provide nonspecialists with additional information in the structured seminars. When the 2 groups were compared, no significant differences between them were observed, either with the SR or with the DA. Although this indicated that the potential effect of experience had been controlled for during our study, it is not possible to say whether this resulted from our differential approach to participant briefing, as this was not an outcome that was measured during our study.

Additionally, we were able to control successfully for potential confounders by randomizing both the order of the questions and the tools used by participants during the vignettes. This was used to address the possibility that participant scores may have improved over time and that the clinical vignettes may not have been completely balanced in their difficulty. Our sensitivity analysis showed that the distribution of questions and tools remained balanced, even after exclusion of participants, and that increasing participant experience with the questionnaire did not result in higher scores.

### Limitations

While the vignettes intentionally challenged participants to navigate the breadth of the decision tables, only a limited number of scenarios are typically encountered in clinical practice. More than 95% of patients will be classified in the lowest risk category for screening based on family history, while most colonoscopies in Australia will detect few or no significant lesions [1]. Thus, participant performance in this study may not be indicative of real-world application. However, adherence rates to current surveillance guidelines, which have been reported at 50.8%, closely resemble the scores obtained using the SR in our study [4]. Clarification of the real-world efficacy of the DA will require further studies, for example, through prospective randomized nested case-controlled studies involving both primary and specialist group practices.

Our study was also prone to sampling bias. Despite efforts to circulate advertising material for the study via social media, education providers, and hospitals, only 117 participants visited the questionnaire website, and the recruitment rate was slow. The diversity of our sample group was also affected, with surgeons outnumbered by gastroenterologists in the specialist group (2-35). Due to our specific subject matter, it is possible that our participants held favorable views toward technology that may not be representative of the greater community of medical professionals. Although these challenges are not uncommon among studies recruiting clinical personnel as participants, the generalizability of our findings may be limited [21].

Finally, as both resources were readily available for public access at the time of the study, it was not possible to restrict participants to using the tools in the prerandomized order specified. Our intention-to-treat analysis may therefore have underestimated the potential differences in the scores obtained by users in the trial.

### Conclusions

Australia’s bowel cancer screening and surveillance guidelines have become increasingly complex, posing a challenge for clinicians trying to make appropriate recommendations. Currently, the available options to assist them are costly and need more scalability. DAs represent an inexpensive and scalable solution that enhances guideline concordance among clinicians. Further development and assessment of these tools could improve screening and surveillance outcomes and optimize resource use in an era of increasingly complex and personalized care.

## Appendix

Multimedia Appendix 1 CONSORT checklist.

## Abbreviations

DA: digital application
NHMRC: National Health and Medical Research Council
REDCap: Research Electronic Data Capture
SR: standard resource
SUS: System Usability Scale

## Data Availability

The data sets generated during and/or analyzed during this study are available from the corresponding author on reasonable request.
